# A study on the changing trend and influencing factors of hospitalization costs of schizophrenia in economically underdeveloped areas of China

**DOI:** 10.1038/s41537-023-00331-6

**Published:** 2023-01-19

**Authors:** Jianjian Li, Hongmei Du, Feng Dou, Chao Yang, Yini Zhao, Zhibin Ma, Xiaobin Hu

**Affiliations:** Department of Epidemiology and Health Statistics, School of Public Health, Lanzhou University, 730000 Lanzhou, Gansu Province China

**Keywords:** Psychology, Schizophrenia, Psychosis

## Abstract

The public health problems caused by schizophrenia are becoming increasingly prominent and can place a huge economic burden on society. This study takes Gansu Province as an example to analyze the level and changing trend of the economic burden of schizophrenia inpatients in economically underdeveloped areas of China. Using a multi-stage stratified cluster sampling method, 39,054 schizophrenics from 197 medical and health institutions in Gansu Province were selected as the research objects, and their medical expenses and related medical records were obtained from the medical information system. The rank sum test and Spearman rank correlation were used for univariate analysis. Quantile regression and random forest were used to analyze the influencing factors. The results show that the average length of stay of schizophrenics in Gansu Province of China was 52.01 days, and the average hospitalization cost was USD1653.96 from 2014 to 2019. During the six years, the average hospitalization costs per time decreased from USD2136.85 to USD1401.33. The average out-of-pocket costs per time decreased from USD1238.78 to USD267.68. And the average daily hospitalization costs increased from USD38.18 to USD41.25. The main factors influencing hospitalization costs are length of stay, proportion of medications, and schizophrenic subtype. The hospitalization costs per time of schizophrenics in Gansu Province have decreased but remain at a high level compared to some other chronic non-communicable diseases. In the future, attention should be paid to improving the efficiency of medical institutions, enhancing community management, and promoting the transformation of the management model of schizophrenia.

## Introduction

Schizophrenia is a complex psychiatric disorder, often accompanied by obstacles in perception, thinking, emotion, and behavior^1^. And it remains one of the most expensive mental illnesses in human society^2^. The disease occurs in late adolescence and early adulthood, and has a poor prognosis^3^. The mortality rate is 3.12 times higher in the patient population than in the general population^4^. It is reported that the number of schizophrenics worldwide increased from 13.1 million in 1990 to 20.9 million in 2016^3^. The global age-standardized prevalence of schizophrenia is 287.4 per 100,000 in 2019^5^. And the lifetime prevalence in China is 0.6%^6^. The public health problems caused by schizophrenia have become increasingly prominent because of the concentration of the disease in young adults, the high unemployment rate of patients^7^, the susceptibility to co-morbidity with other somatic diseases^8^, and the high incidence of aggression and violence^9^. And they also bring huge economic burden to schizophrenic families and the society.

The economic burden of illness is measured and analyzed in terms of the economic costs or losses incurred by the population due to the disease, including the costs of treatment and care for the patients, as well as the loss of productivity due to injury, disability, caregiver assistance or the patient’s violent behavior^10^. It mainly includes direct economic burden, indirect economic burden, and intangible economic burden. The direct economic burden reflects the medical resources used in the course of disease treatment^11^, and the hospitalization expense is the largest contributor to the total direct medical costs of schizophrenia^12^. The existing research shows that the social costs due to schizophrenia vary widely globally^13^. The global lifetime social cost of schizophrenia per capita ranges from USD5818 in Thailand to USD94,587 in Norway^14^. The level of economic development and medical care varies greatly among different regions in China, therefore the level of the economic burden of this disease may also vary greatly. However, only small sample data or medical insurance data have been reported in China to analyze the economic burden of the illness^15–17^, and they are mostly concentrated in more economically developed regions. Few relevant studies using large sample treatment data in less economically developed regions have been seen.

Gansu Province is located in an economically underdeveloped region in northwestern China, with a resident population of 26.47 million at the end of 2019. The GDP of the province this year was 871.83 billion Chinese yuan, with 12 prefecture level cities and 2 autonomous prefectures under its jurisdiction^18^. At present, there is a shortage of full-time prevention and control personnel of mental health in the province. In 2018, the standardized management rate of registered patients with severe mental disorders was 71.78%, of which the regular medication-taking rate of schizophrenia was only 27.49%^19^. Therefore, this study using Gansu Province as an example has more research significance in analyzing the level of economic burden of schizophrenia in economically underdeveloped regions of China. This study used the treatment data of schizophrenia inpatients to analyze the composition and changing trend of hospitalization costs in Gansu Province of China from 2014 to 2019. At the same time, it explored the factors influencing the average hospitalization cost per time and the average daily hospitalization cost of schizophrenics and their significance. Thus, it provides a theoretical basis for policy-makers to formulate relevant health policies and reduce the economic burden of schizophrenia.

## Results

### Patient characteristics and univariate analysis

A total of 39,054 inpatients with schizophrenia were enrolled in this study from 2014 to 2019, including 21,272 males (54.47%) and 17,782 females (45.53%). The average age was 37.28 ± 12.32 years old. The average length of stay was 52.01 ± 41.87 days. And the percentage of patients using the medical insurance reimbursement payment method was 59.17%. In terms of institutional level (graded by administrative subordination), 98.25% of patients were treated in provincial or municipal medical and health institutions. In terms of the type of institution, 94.32% of the patients were seen in specialized hospitals. Among schizophrenia inpatients with defined subtypes, paranoid schizophrenia had the highest percentage of 34.06%.

The results of the univariate analysis showed statistically significant differences in the average costs of hospitalization per time between patients with different gender, ages, payment methods, hospital nature, hospital levels, hospital types, number of comorbidities, length of stay, proportion of medications, and schizophrenic subtypes (*P* < 0.05). See Table 1.

**Table 1:** Basic characteristics of patients and univariate analysis of average hospitalization costs per time.

Category	*n* (q%)	Median (lower quartile, upper quartile) (CNY)	Test statistic	*P*
All cases	39,054	11409.83 (6718.96, 16915.64)	–	–
Gender	–	–	−15.396	<0.001
Male	17,782 (45.53)	10774.17 (6469.78, 15706.02)	–	–
Female	21,272 (54.47)	12060.85 (6899.51, 17843.94)	–	–
Age (years) [mean(SE)]	37.28 (12.32)	–	−0.123	<0.001
Payment method	–	–	−30.018	<0.001
Medical insurance reimbursement	23,107 (59.17)	10151.70 (5896.50, 16286.45)	–	–
Out-of-pocket	15,947 (40.83)	12806.77 (8337.56, 17603.35)	–	–
Hospital nature	–	–	6.420	<0.001
Public	38,762 (99.25)	11448.53 (6718.82, 16962.42)	–	–
Private	292 (0.75)	9795.69 (6789.42, 12038.91)	–	–
Hospital level	–	–	15.935	<0.001
Provincial and municipal level	38,370 (98.25)	11,513 (6766.90, 16988.19)	–	–
District and county level and below	684 (1.75)	7130.61 (2740.46, 11795.34)	–	–
Hospital type	–	–	1768.391	<0.001
General hospital	2001 (5.12)	4853.51 (4281.12, 6825.58)	–	–
Specialized hospital	36,835 (94.32)	11844.33 (7032.98, 17159.87)	–	–
Traditional Chinese Medicine (TCM) hospital	55 (0.14)	2990.21 (1894.69, 4420.25)	–	–
Primary medical and health institution^a^	163 (0.42)	11880.85 (5899.37, 19894.22)	–	–
Number of comorbidities	–	–	1734.708	<0.001
0	17,788 (45.55)	9615.51 (5462.09, 15122.00)	–	–
1	1850 (4.74)	11593.72 (6831.93, 15406.83)	–	–
2	17,635 (45.16)	12839.54 (7401.57, 18030.52)	–	–
≥3	1781 (4.56)	16098.10 (11001.90, 21932.16)	–	–
Length of stay (days) [mean(SE)]	52.01 (41.87)	–	0.725	<0.001
Proportion of medications (%) [mean(SE)]	19.91 (22.20)	–	−0.303	<0.001
Schizophrenic subtype	–	–	8128.980	<0.001
Paranoid schizophrenia	13,303 (34.06)	14471.85 (10507.08, 18756.91)	–	–
Hebephrenic schizophrenia	7 (0.02)	20030.60 (2383.90, 21461.96)	–	–
Catatonic schizophrenia	56 (0.14)	12436.66 (9220.39, 17801.39)	–	–
Undifferentiated schizophrenia	7027 (17.99)	14920.70 (10710.49, 19780.56)	–	–
Post-schizophrenic depression	177 (0.45)	2965.03 (1750.69, 5656.85)	–	–
Residual schizophrenia	129 (0.33)	23103.14 (15912.86, 27908.42)	–	–
Simple schizophrenia	9 (0.02)	10325.25 (7888.11, 13843.42)	–	–
Other schizophrenia	2 (0.01)	8311.86 (5984.29, 10639.42)	–	–
Schizophrenia, unspecified	18,344 (46.97)	7234.94 (4658.88, 12244.51)	–	–

^a^Primary medical and health institutions include township health centers and community health service centers.

### Component of hospitalization costs for schizophrenics

**Fig. 1:**
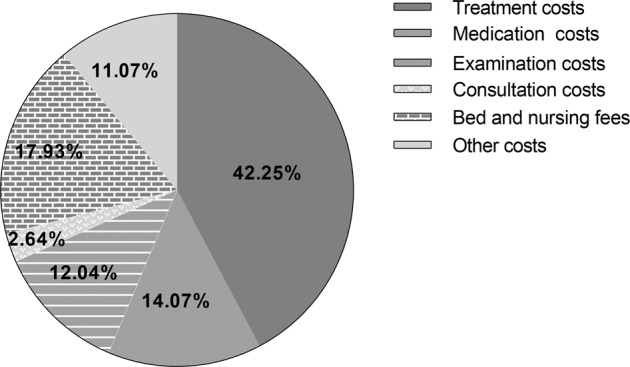
Composition of hospitalization expenses of schizophrenic patients. Composition of hospitalization costs of schizophrenia patients in Gansu Province, China, from 2014 to 2018.

From 2014 to 2019, the average hospitalization expenses of schizophrenics per visit in Gansu Province were CNY11,409.83 (USD1653.96). Among them, the treatment expenses were CNY4,568.64 (USD662.27, 42.25%), which accounted for the highest percentage. This was followed by bed and nursing fees of CNY1938.65 (USD281.02, 17.93%), medication costs of CNY1521.65 (USD220.58, 14.07%), and examination costs of CNY1301.55 (USD188.67, 12.04%). The proportion of consultation costs accounted for the lowest, at CNY286.00 (USD41.46, 2.64%). See Fig. 1.

### The trend of hospitalization costs for schizophrenics

From 2014 to 2019, the average hospitalization costs of schizophrenics per time in Gansu Province showed an overall downward trend, from CNY14,741.08 (USD2136.85) to CNY9667.07(USD1401.33; ***r***_***s***_ = −0.161, *P* < 0.001). The average growth rate over the 6 years was −8.09%. Average inpatient out-of-pocket costs have also been decreasing from CNY8545.74 (USD1238.78) in 2014 to CNY1846.56 (USD267.68) in 2019 (***r***_***s***_ = −0.294, *P* < 0.001). The average growth rate was −26.39%. However, during the same period, the average daily hospitalization costs for patients showed an overall increasing trend from CNY263.41 (USD38.18) to CNY284.56 (USD41.25), with an average growth rate of 1.56%. See Table 2.

**Table 2:** Trends of hospitalization expenses of schizophrenics.

Year	Hospitalization costs (CNY)^a^	Growth rate (%)^b^	Out-of-pocket costs (CNY)^a^	Growth rate (%)	Length of stay (days)	Daily hospitalization costs (CNY)	Growth rate (%)
2014	14741.08	–	8545.74	–	58.00	263.41	–
2015	12370.95	−16.08^b^	6391.71	−25.21	52.00	280.32	6.42
2016	11546.41	−6.67	4792.08	−25.03	45.00	287.12	2.42
2017	10573.83	−8.42	4162.13	−13.15	41.00	287.74	0.22
2018	9222.21	−12.78	2468.32	−40.70	36.00	278.33	−3.27
2019	9667.07	4.82	1846.56	−25.19	38.00	284.56	2.24
Overall^c^	11409.83	−8.09^c^	4146.81	−26.39	44.00	280.28	1.56
*r*^*s*^	−0.161	–	−0.294	–	–	0.056	–
*P*	<0.001	–	<0.001	–	–	<0.001	–

^a^The above expenses refer to average hospitalization costs per time.

^b^The growth rate of each year is the year-on-year growth rate based on the previous year.

^c^The overall growth rate is the average growth rate from 2014 to 2019.

### Quantile regression analysis of hospitalization costs

Natural log-transformed hospitalization costs per time and average daily hospitalization costs of schizophrenia patients were used as dependent variables. Variables that were statistically significant for univariate analysis, such as gender, age, payment method, hospital nature, hospital level, hospital type, number of comorbidities, length of stay, proportion of medications, schizophrenic subtype, and year of hospitalization, were used as independent variables. Incorporating each of the above variables into the quantile regression model simultaneously. The Pseudo *R*^2^ of the model in the 10th, 50th, and 90th percentile points of hospitalization costs were 0.479, 0.529, and 0.542 respectively, with the best fit at the 90th percentile. The Pseudo *R*^2^ at the three percentile points of average daily hospitalization costs were 0.637, 0.287, and 0.156 respectively, with the best fit at the 10th percentile. The results showed that the effects of age, payment method, hospital nature, hospital level, length of stay, and proportion of medications were significant at all three percentile levels of hospitalization costs and average daily hospitalization costs (all *P* < 0.05).

Age, hospital level, and proportion of medications had a significant negative effect (*P* < 0.05) on all three quantile points of hospitalization costs per time, and the intensity of the effect was higher at the lower quantile point than at the higher point. Length of stay was positively associated with hospitalization costs, with the strength of its effect being slightly higher at the higher quartile point than at the lower. Among payment methods, full out-of-pocket patients had lower hospitalization costs at the 10th percentile and higher at the remaining two quartiles than medical insurance reimbursement patients (all *P* < 0.05). For the number of comorbidities, patients with one, two, and three or more comorbidities had higher hospitalization costs at the 50th and 90th percentiles than patients without comorbidities (all *P* < 0.05).

Hospital nature, length of stay, and proportion of medications had a significant negative effect at all three quartiles of average daily hospitalization costs (all *P* < 0.05). In contrast, payment method and hospital level had a significant positive impact on the three quantiles (all *P* < 0.05). For the number of comorbidities, patients with one, two, and three or more comorbidities had higher average daily hospitalization costs at the 50th and 90th percentiles than patients without comorbidities (all *P* < 0.05). See Table 3.

**Table 3:** Quantile regression analysis of hospitalization costs in schizophrenics (CNY).

Variables	Hospitalization costs per time			Average daily hospitalization costs		
	Q10	Q50	Q90	Q10	Q50	Q90
Gender (contrast = male)						
Female	−0.013	−0.018^**^	0.008^*^	−0.038^**^	−0.023^**^	−0.009
Age	−0.004^**^	−0.003^**^	−0.002^**^	−0.002^**^	−0.001^**^	0.001^**^
Payment method (contrast = medical insurance reimbursement)						
Out-of-pocket	−0.044^**^	0.053^**^	0.044^**^	0.073^**^	0.074^**^	0.089^**^
Hospital nature (contrast = public)						
Private	0.557^**^	0.158^**^	−0.079^*^	−1.051^**^	−1.064^**^	−1.090^**^
Hospital level (contrast = provincial and municipal level)						
District and county level and below	−0.814^**^	−0.341^**^	−0.175^**^	0.788^**^	0.683^**^	0.631^**^
Hospital type (contrast = general hospital)						
Specialized hospital	0.318^**^	0.212^**^	0.074^**^	0.346^**^	0.101^**^	−0.262^**^
TCM hospital	−0.448^*^	0.080	0.170^*^	−0.296^*^	−0.023	−0.172^*^
Primary medical and health institution	0.912^**^	0.805^**^	1.283^**^	−0.550^**^	−0.104^*^	−0.060
Number of comorbidities (contrast = 0)						
1	0.043	0.049^**^	0.064^**^	0.005	0.035^**^	0.061^**^
2	0.055^*^	0.129^**^	0.184^**^	0.042^**^	0.083^**^	0.074^**^
≥3	0.107^**^	0.245^**^	0.248^**^	0.164^**^	0.188^**^	0.173^**^
Length of stay	0.010^**^	0.013^**^	0.013^**^	−0.005^**^	−0.004^**^	−0.004^**^
Proportion of medications	−0.026^**^	−0.020^**^	−0.012^**^	−0.027^**^	−0.019^**^	−0.007^**^
Subtype (contrast = paranoid schizophrenia)						
Hebephrenic schizophrenia	−0.259	0.058	0.195	−0.099	0.188	0.841^**^
Catatonic schizophrenia	0.048	0.041	0.059	0.046	0.076	0.016
Undifferentiated schizophrenia	−0.003	−0.018^*^	−0.007	−0.009	0.004	0.006
Post-schizophrenic depression	−0.654^**^	−0.393^**^	−0.147^**^	−0.570^**^	−0.356^**^	−0.085^*^
Residual schizophrenia	−0.046	−0.206^**^	−0.130^**^	−0.283^**^	−0.021	0.148^*^
Simple schizophrenia	−0.178	0.091	0.537^**^	−0.344	0.237	0.129
Other schizophrenia	−0.281	−0.468	−0.242	0.223	0.063	0.141
Schizophrenia, unspecified	−0.471^**^	−0.212^**^	−0.099^**^	−0.320^**^	−0.103^**^	−0.028^**^
Year (contrast = 2014)						
2015	0.024	0.168^**^	0.218^**^	0.076^**^	0.137^**^	0.138^**^
2016	0.019	0.120^**^	0.177^**^	0.039^*^	0.094^**^	0.104^**^
2017	0.029	0.169^**^	0.229^**^	0.082^**^	0.144^**^	0.168^**^
2018	−0.044^*^	−0.033^**^	−0.006	−0.015	−0.018^*^	0.096^**^
2019	0.002	−0.034^**^	0.037^**^	−0.014	−0.001	0.082^**^
Pseudo *R*^2^	0.479	0.529	0.542	0.637	0.287	0.156

^*^P < 0.05, ^**^P < 0.001.

### Analysis of the importance of factors influencing hospitalization costs

Natural log-transformed hospitalization costs per time and average daily hospitalization costs of schizophrenia patients were used as dependent variables. Factors from the quantile regression analysis that had a significant effect on hospitalization costs were used as independent variables. Incorporating them jointly into the random forest regression tree model. The training set and testing set sample cases are randomly assigned in the ratio of 3:1. Set the number of random seed to 666, the number of trees to grow (ntree) to 500, and the number of variables randomly sampled as candidates at each split (mtry) to 3. At this point, the standardized mean squared error (RMSE) of the random forest regression tree model for hospitalization costs per time was 0.266 with *R*^2^ = 0.898, and the RMSE for average daily hospitalization costs was 0.257 with *R*^2^ = 0.857. The model generalization ability is strong and the fit is excellent.

**Fig. 2:**
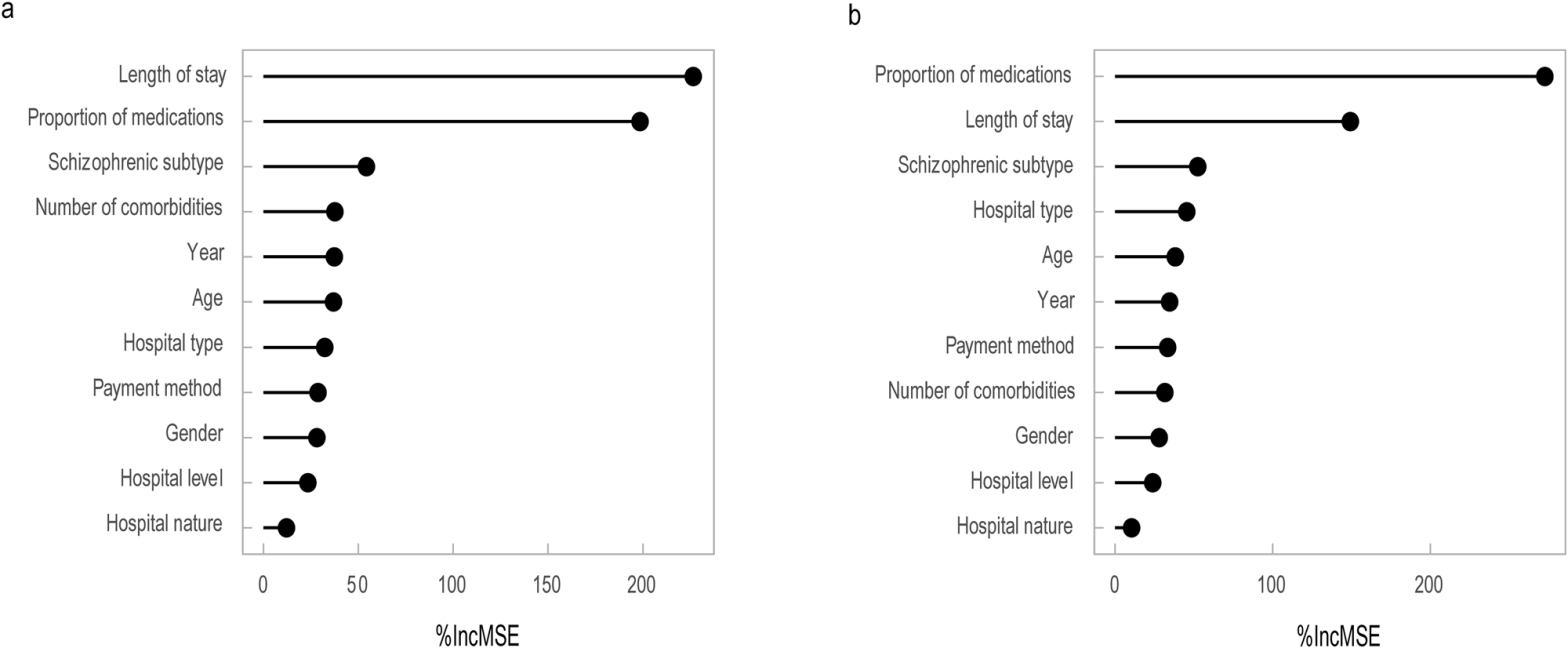
Analysis on the importance of influencing factors of hospitalization expenses of schizophrenics. **a** Ranking the importance of factors on the hospitalization costs per time of patients with schizophrenia. **b** Ranking the importance of factors on the average daily hospitalization costs of patients with schizophrenia.

Finally, the importance of the influencing factors was analyzed according to the mean decrease accuracy (%IncMSE). That is, the larger the %IncMSE, the higher the importance. The results showed that the top five influencing factors for hospitalization costs per time were length of stay, proportion of medications, schizophrenic subtype, number of comorbidities, and year of hospitalization, respectively. The top five factors influencing average daily hospitalization costs were proportion of medications, length of stay, schizophrenic subtype, hospital type, and age. See Fig. 2.

## Discussion

This study analyzed the hospitalization costs of schizophrenia patients in economically underdeveloped areas of China, using a large sample of consecutive 6 years of treatment data in Gansu Province. The results showed that the average age of schizophrenia inpatients in Gansu Province from 2014 to 2019 was 37.28 ± 12.32 years, and the average length of stay was 52.01 ± 41.87 days. Previous studies have shown that the average length of stay for patients with schizophrenia in Central and Eastern Europe (CEE) and the United States was 25.3 and 9.08 days, respectively^10,20^. The median length of stay for schizophrenia inpatients in Portugal was 18.0 days, and the mean age of the patients was 41.68 ± 12.93 years^21^. In comparison, Chinese patients with schizophrenia were hospitalized at a younger age and for a longer length of stay. This may be because countries such as the United States have implemented integrated community-based rehabilitation interventions, which have shifted from an inpatient model to a community-based model for schizophrenia services, thereby the length of stay has been significantly reduced^22^. Medical resources are scarce in underdeveloped areas in northwest China. And there are few full-time mental health prevention and control personnel in grassroots institutions. It is difficult for patients to obtain high-quality community nursing services after discharge. The above together lead to the longer length of stay for schizophrenia in China. The average hospitalization costs per visit of schizophrenia patients in Gansu Province from 2014 to 2019 was CNY 11,409.83 (USD 1653.96). A higher percentage of these are for treatment costs, bed and nursing fees, and medication costs, with the three accounting for more than 80% of the total costs. The average hospitalization costs per time for schizophrenia is lower than that of gastric cancer (CNY12,514.98 in 2017)^23^, but much higher than some other chronic non-communicable diseases such as diabetes (CNY 5131.34 in 2018) and coronary heart disease (CNY 6791.38 in 2015)^24,25^. Unlike the medication-based cost model for non-psychiatric disorders, treatment costs, which measure the value of the medical workforce, account for the highest proportion of schizophrenia hospitalization costs. This is consistent with the findings of other similar schizophrenia studies^15^.

The results showed that the average hospitalization costs and out-of-pocket costs per time of schizophrenia patients in Gansu Province showed a decreasing trend from 2014 to 2019, with an average growth rate of −8.09% and −26.39% respectively. The decrease in hospitalization costs may be related to the promotion of graded treatment in the region and the improvement of service capacity of primary medical and health institutions. The region has implemented the service model of “treating schizophrenia in the hospital and managing rehabilitation in the community”. For patients in the acute stage and unstable condition, the primary institutions promptly refer them to professional mental institutions for standardized treatment. After stabilization, they return to the community to receive maintenance treatment with basic psychiatric medicines^26^. Average out-of-pocket costs per time declined obviously faster than hospitalization costs. This may be related to the comprehensive promotion of rescue and treatment assistance for severe mental disorders and the gradual improvement of medical coverage level for the patients in the region. The average daily hospitalization costs showed an overall increasing trend over the 6-year period, with an average rate of increase of 1.56%. This was driven primarily by the decrease in length of stay.

Random forest is an integrated learning algorithm based on decision trees, which has better tolerance for outliers and noise and is less prone to overfitting^27,28^. This study used random forest regression tree model to obtain the importance ranking of independent variables on the degree of impact of hospitalization costs. The results showed that length of stay, medication proportion, and schizophrenic subtype are the main factors influencing the hospitalization costs. Combined with the quantile regression results, the average hospitalization cost per time was negatively correlated with age and medication proportion, and positively correlated with length of stay and number of comorbidities. A previous Chinese study showed that the optimal length of stay for functional recovery in schizophrenics was between 20 and 50 days^29^. Therefore, the length of stay for schizophrenia patients should be reasonably reduced and other physical illnesses of the patients should be actively prevented. The efficiency of treatment in medical institutions should also be continuously improving. This will, on the one hand, help to increase bed turnover and bring more economic benefits to medical institutions, and on the other hand, help to reduce the patient’s medical costs. The average cost of a hospital stay at district and county level is lower than at provincial and municipal level. And the cost of specialized hospitals is higher than that of general hospitals. The number of inpatients with schizophrenia attending provincial and municipal level medical institutions is much higher than that of district and county level. This may be due to the fact that higher-grading institutions are better equipped and have a higher level of diagnosis and treatment. Therefore, patients are more likely to visit such facilities, and the costs are correspondingly higher. The hospitalization costs for full out-of-pocket patients are higher than for medical insurance reimbursement patients. This suggests that medical insurance has had a cost-controlling effect on schizophrenia inpatients. It has reduced unnecessary expenditure in medical costs, thus having a positive effect on reducing the economic burden of the disease.

This study also has some limitations. Firstly, the data for the study were obtained from the billing and settlement systems of healthcare institutions. Hence the variables included were somewhat limited and could not include other variables such as disease severity that may affect hospitalization costs. Second, for reasons such as the poor implementation of schizophrenia typing in Chinese clinical practice, some of the available statistics do not contain detailed information on disease subtypes. This may limit the further mining of data information.

In conclusion, the average costs per time and the length of stay of schizophrenia inpatients in Gansu Province, China, decreased from 2014 to 2019, but currently remain at a high level. Therefore, in order to further reduce the level of the patient’s economic burden, the length of stay should be reasonably reduced. At the same time, the treatment efficiency of medical institutions should be improved, and the capacity of primary medical and health institutions for prevention and control of mental illness should be strengthened. Thus, the community management level of schizophrenia can be further improved and the rational distribution of medical resources can be continuously promoted.

## Methods

### Data source

In this study, a multi-stage stratified cluster sampling method was used. In stage 1, among the provincial institutions, half of the number of general hospitals and Traditional Chinese Medicine hospitals (TCM hospitals) and one of each different type of specialized hospitals were selected as sample institutions using simple random sampling method. In stage 2, based on the economic level, geographical condition, and population size of the 14 cities and prefectures in Gansu Province, five cities, namely Pingliang, Dingxi, Zhangye, Wuwei, and Tianshui, were finally selected as sample cities. The cities follow the same sampling principle as the provincial medical and health institutions for municipal institutions. In stage 3, according to the geographical location and the urban and rural characteristics, one district and two counties were selected in each sample city, totaling 15 counties (districts). And then according to the provincial institutions sampling principle, the county (district) level medical and health institutions shall be selected. In stage 4, among the selected counties (districts), 5–8 township health centers or community health service centers were selected by simple random sampling method. Eventually, a total of 197 medical and health institutions were acquired.

According to the code F20 of the International Classification of Diseases 10th Revision (ICD-10) issued by WHO, the medical records of inpatients with a primary diagnosis of schizophrenia were collected in each sample institution from 2014 to 2019. Among them, the ICD-10 code F20.0 refers to paranoid schizophrenia. F20.1 refers to hebephrenic schizophrenia. F20.2 refers to catatonic schizophrenia. F20.3 refers to unidentified schizophrenia. F20.4 refers to post-schizophrenic depression. F20.5 refers to residual schizophrenia. F20.6 refers to simple schizophrenia. F20.8 refers to other schizophrenia. F20.9 refers to schizophrenia, unspecified. We obtain the patient’s case information, including general demographic information (age, gender, disease diagnosis, date of discharge, length of stay, and information about the medical institution, etc.) and hospitalization cost information (treatment costs, medication costs, examination costs, consultation costs, bed and nursing fees, and other costs). Finally, a database of hospitalization costs of schizophrenia patients was formed.

### Data pre-processing

Hospitalization costs are the costs incurred by the patient for the diagnosis and treatment of the disease during hospitalization. Among them, medication costs include TCM costs and western medicine costs. Examination costs include check fees, test fees, and radiate fees. Bed and nursing fees include bed fees and nursing fees. Other costs include all expenses except treatment costs, medication costs, examination costs, consultation costs, and bed and nursing fees. Proportion of medications refers to the proportion of medication costs to the total hospitalization costs.

After data entry, the integrity and authenticity of the data were verified and the logical error correction was performed promptly. Subsequently, the information with incorrect ICD-10 coding should be corrected. Reject the unqualified data, including: (1) Primary diagnosis of non-schizophrenia. (2) Missing and poorly documented major items. (3) Logical relationships between costs that were clearly incorrect and could not be logically corrected. (4) The presence of outlier data. A total of 39,054 hospitalization data for patients with schizophrenia were eventually collected in this study. To ensure comparability between cost data across years, the costs were converted comparably using the GDP deflator for 2019 as the base year. The relevant calculation formula is shown below Eqs. (1) (2). The nominal GDP values and GDP indices for each year are obtained from the official data of the National Bureau of Statistics of the People’s Republic of China. According to data from the People’s Bank of China, the annual exchange rate between the U.S. dollar and the Chinese yuan for 2019 is USD1.0 = CNY6.8985.

1 $${{{\mathrm{GDP}}}}\;{{{\mathrm{deflator}}}} = \frac{{{{{\mathrm{Nominal}}}}\;{{{\mathrm{GDP}}}}\;{{{\mathrm{of}}}}\;{{{\mathrm{the}}}}\;{{{\mathrm{year}}}}}}{{{{{\mathrm{Nominal}}}}\;{{{\mathrm{GDP}}}}\;{{{\mathrm{in}}}}\;{{{\mathrm{base}}}}\;{{{\mathrm{period}}}} \times ({{{\mathrm{GDP}}}}\;{{{\mathrm{index}}}}\;{{{\mathrm{of}}}}\;{{{\mathrm{the}}}}\;{{{\mathrm{year}}}}/{{{\mathrm{GDP}}}}\;{{{\mathrm{index}}}}\;{{{\mathrm{in}}}}\;{{{\mathrm{base}}}}\;{{{\mathrm{period}}}})}}$$

2 $${{{\mathrm{Real}}}}\;{{{\mathrm{hospitalization}}}}\;{{{\mathrm{costs}}}} = \frac{{{{{\mathrm{Nominal}}}}\;{{{\mathrm{hospitalization}}}}\;{{{\mathrm{costs}}}}}}{{{{{\mathrm{GDP}}}}\;{{{\mathrm{deflator}}}}}}$$

### Statistical analysis

Excel 2016 was used to standardize the data obtained from the field survey. Stata 14.0 and R 4.1.3 software were used to build the database and perform data cleaning and statistical analysis. And GraphPad Prism 8.3.0 and R 4.1.3 software were used to make graphs. Qualitative data were expressed as number of cases (composition ratio) [n(q%)], and measurement data were expressed as mean ± standard deviation [mean(SE)]. Since hospitalization costs are positively skewed, the median and quartiles [M(P_25_, P_75_)] were used to describe their distribution. Univariate analysis of average hospitalization costs per time of each classification of patients using Mann–Whitney *U* test and Kruskal–Wallis *H* test. Spearman’s rank correlation was used to analyze the correlation between non-normally distributed hospitalization costs and age, length of stay, proportion of medications, and year. Transforming the hospitalization costs into a natural logarithm so that it follows an approximately normal distribution. The factors influencing the hospitalization costs and their importance were analyzed using quantile regression model and random forest regression tree model. In this study, based on the experience of previous similar studies^30^, the 10th, 50th, and 90th percentiles of hospitalization costs were selected for fitting in the quantile regression model. This provides a comprehensive picture of the effect of independent variables on low, medium, and high levels of hospitalization costs. Variable assignment is shown in Table 4. The two-sided test level *α* = 0.05.

**Table 4:** Variable assignment of quantile regression and random forest model.

Variables	Variable assignment
Gender	1 = Male, 2 = Female
Age (years)	Actual value
Payment method	1 = Medical insurance reimbursement, 2 = Out-of-pocket
Hospital nature	1 = public, 2 = private
Hospital level	1 = Provincial and municipal level, 2 = District and county level, and below
Hospital type	1 = General hospital, 2 = Specialized hospital, 3 = TCM hospital, 4 = Primary medical and health institution
Number of comorbidities	0 = 0, 1 = 1, 2 = 2, 3 = 3 or more
Length of stay (days)	Actual value
Proportion of medications (%)	Actual value
Schizophrenic subtype	1 = Paranoid schizophrenia, 2 = Hebephrenic schizophrenia, 3 = Catatonic schizophrenia, 4 = Undifferentiated schizophrenia, 5 = Post-schizophrenic depression, 6 = Residual schizophrenia, 7 = Simple schizophrenia, 8 = Other schizophrenia, 9 = Schizophrenia, unspecified
Year^a^	1 = 2014, 2 = 2015, 3 = 2016, 4 = 2017, 5 = 2018, 6 = 2019
Average hospitalization costs per time	Ln (actual value)
Average daily hospitalization expenses	Ln (actual value)

^a^The year is based on the patient’s discharge time in the medical record system.

## Data Availability

The data are not publicly available at present. However, it can be obtained from the corresponding author according to reasonable requirements.
